# Anthracycline-containing versus anthracycline-free carboplatin-based regimens in early-stage triple-negative breast cancer: real-world outcomes

**DOI:** 10.1007/s10549-026-08071-8

**Published:** 2026-09-09

**Authors:** Matthew R. Kroll, Mary C. Schroeder, Joan M. Neuner, Brahmendra R. Viyyuri, Aditya Ravindra, Dana McDougall, Cole G. Chapman, Nicole M. G. Fleege, Elizabeth A. Chrischilles, Xing Song, Sneha Phadke

**Affiliations:** 1 Division of Hematology, Oncology, and Blood & Marrow Transplantation, Department of Internal Medicine, University of Iowa Carver College of Medicine, Iowa City, IA USA; 2 Division of Health Services Research, Department of Pharmacy Practice and Science, University of Iowa College of Pharmacy, Iowa City, IA USA; 3 Division of General Internal Medicine, Department of Medicine, University of Wisconsin School of Medicine and Public Health, Madison, WI USA; 4 MercyOne Waterloo Hematology/Oncology, Waterloo, IA USA; 5 Department of Epidemiology, University of Iowa College of Public Health, Iowa City, IA USA; 6 Department of Biomedical Informatics, Biostatistics and Medical Epidemiology, University of Missouri School of Medicine, Columbia, MO USA

**Keywords:** Triple-negative breast cancer, Complete response, Neoadjuvant chemotherapy, Carboplatin, Anthracyclines

## Abstract

**Purpose:**

Triple‑negative breast cancer (TNBC) is an aggressive disease with poor survival outcomes. With the integration of immunotherapy, the optimal neoadjuvant chemotherapy backbone remains poorly defined. We examined real-world treatment patterns and response rates among patients receiving a carboplatin-based regimen, with or without anthracyclines.

**Methods:**

In this retrospective cohort study, women diagnosed 2012–2024 with stage I-III TNBC who received neoadjuvant carboplatin plus taxane (CbT) or CbT with anthracyclines (AC-CbT) were identified from electronic health records data from 10 sites within the Greater Plains Collaborative network. Registry-recorded physician assessment of pathologic complete response (pCR) was the primary outcome. Multivariable logistic regression evaluated whether adding anthracyclines to carboplatin-based treatment was associated with pCR.

**Results:**

Among 878 patients with TNBC, 41.2% received CbT and 58.8% received AC-CbT. Overall, 46% received pembrolizumab, 11.7% in the CbT group and 66.3% in the AC-CbT group. On univariate analysis, pCR rates did not differ between the CbT and AC-CbT groups (47.2% vs. 51.9%, OR = 1.21, 95%CI 0.92–1.58), among patients who underwent surgery. In multivariate adjusted analyses, AC-CbT was not associated with higher odds of pCR (aOR = 1.05, 95%CI 0.69–1.59), among patients who underwent surgery. Other patient characteristics associated with pCR included pembrolizumab use, higher-grade disease, lower stage, and age at diagnosis (40–49 versus 60+). Use of carboplatin-containing regimens increased substantially over time.

**Conclusion:**

In this real-world cohort, addition of anthracycline to carboplatin-containing regimen was not associated with an improvement in pCR rates. These findings suggest clinical equipoise regarding anthracycline omission in early-stage TNBC, to be validated in prospective trials. Additional work evaluating adherence, toxicity, and long-term outcomes is needed.

**Supplementary Information:**

The online version contains supplementary material available at https://doi.org/10.1007/s10549-026-08071-8.

## Introduction

Triple-negative breast cancer (TNBC), defined by the absence of expression of estrogen receptor (ER) and progesterone receptor (PR) on immunohistochemistry (IHC), and non-pathologic human epidermal growth factor receptor 2 (HER2) expression on IHC or in situ hybridization (ISH), represents approximately 10–15% of all breast cancers and is associated with a disproportionately high risk of early recurrence and mortality [1, 2]. The aggressive biology and clinical heterogeneity of TNBC have historically limited non-cytotoxic systemic treatment options, making multiagent cytotoxic chemotherapy the backbone of early-stage management. Among these systemic therapies, anthracycline-and-taxane-based regimens have long served as the standard of care [3]. These regimens historically produce pathologic complete response (pCR) rates of 37–41% [2]. pCR is a strong predictor of survival outcomes at the patient level, with 5-year event-free survival (EFS) reaching 90% in patients who achieve it versus (vs.) 57% in those who do not [4]. However, the use of anthracycline in this regimen exposes patients to the risks of late cardiotoxicity and secondary malignancies [5–10]. Pooled data from randomized controlled trials report a larger than five-fold higher risk of both clinical cardiotoxicity [11] and acute myeloid leukemia and myelodysplastic syndrome [6] with anthracycline compared with non-anthracycline regimens.

The incorporation of carboplatin into neoadjuvant and adjuvant chemotherapy regimens has made therapeutic decision-making in TNBC more complex. While several studies demonstrate that carboplatin increases the pCR rate, its routine use has been challenged due to added toxicity and inconsistent improvements in long-term survival [12–14]. Recent meta-analyses of randomized controlled trial data indicate improved pCR rate, disease-free survival, and overall survival (OS) with the addition of carboplatin, but at the cost of more treatment delays, dose reductions, and hematologic toxicity [15, 16]. The GeparSixto trial reported Grade 3–4 neutropenia (65% vs. 27%), anemia (15% vs. < 1%), and thrombocytopenia (14% vs. < 1%) and higher incidence of high-grade diarrhea with the addition of carboplatin to anthracycline-containing regimens [14]. The BrighTNess trial also demonstrated that prior carboplatin exposure increased the risk of febrile neutropenia during subsequent cycles of anthracycline-based chemotherapy [12].

The incorporation of immunotherapy to the neoadjuvant treatment backbone introduced an additional paradigm shift. The KEYNOTE-522 trial, which evaluated four cycles of carboplatin and paclitaxel followed by four cycles of anthracycline and cyclophosphamide with and without the addition of pembrolizumab, established perioperative pembrolizumab combined with neoadjuvant chemotherapy as the standard of care for stage II–III TNBC, significantly improving pCR and OS [17, 18]. Yet translating this data into real-world clinical practice revealed challenges in delivering this regimen, including higher than anticipated immune-related adverse events, increased treatment discontinuation, and frequent dose reductions—raising concerns about the tolerability of multidrug regimens that often include both anthracyclines and carboplatin. In a real-world cohort of 100 TNBC patients receiving the KEYNOTE-522 regimen, 52% experienced Common Terminology Criteria for Adverse Events (CTCAE) v5.0 Grade 3–5 neutropenia, 35% discontinued neoadjuvant therapy due to toxicity, and 50% required dose reductions [19]. These findings magnify the need to assess regimen selection in everyday practice in addition to controlled trial settings.

Finally, emerging evidence suggests that carboplatin-based regimens that omit anthracyclines may deliver comparable pCR and short-term EFS rates. Data from the Phase II NeoSTOP and NeoPACT trials report similar treatment responses regardless of anthracycline use [20, 21], and raise the possibility that anthracycline exposure may not be necessary in all patients.

In this evolving therapeutic landscape, a key knowledge gap persists: how do both outcomes and toxicity differ between anthracycline-containing regimens and anthracycline-free, carboplatin-based regimens in real-world patients? Given the restrictive inclusion criteria of clinical trials [22], understanding treatment effectiveness outside controlled environments is essential for informed clinical decision-making. The present study addresses this need by evaluating real-world treatment patterns and outcomes of both anthracycline-containing and anthracycline-free carboplatin-based regimens for early-stage TNBC before and after the immunotherapy era.

## Methods

### Data source

The Greater Plains Collaborative (GPC) is a PCORnet^®^ Clinical Research Network of medical centers serving a diverse population across nine states [23]. GPC provides research data through the GPC Reusable Observable Unified Study Environment (GROUSE), a centralized and de-identified repository that integrates electronic health records (EHR), billing data, and hospital tumor registry data from participating sites [24, 25]. The hospital cancer registry data within GROUSE are collected according to standards developed by the North American Association of Central Cancer Registries (NAACCR) and populate a dedicated tumor table compatible with the PCORnet Common Data Model framework. This table includes all 774 data elements defined in NAACCR Data Standards Version 18, covering demographics, tumor characteristics, treatments, and outcomes.

### Study cohort

GROUSE data from 10 GPC sites with complete tumor and treatment characteristics were used in this study. We queried the tumor tables to identify adult female patients diagnosed 2012–2024 with first, primary, stage I–III solid breast cancer (ICD-O-3 topography codes C50.0–C50.9, morphology codes 8000–8599) of triple-negative subtype, as defined by NAACCR [26], who initiated neoadjuvant chemotherapy of carboplatin plus taxane (CbT) with or without doxorubicin plus cyclophosphamide (AC) within 120 days of diagnosis and received surgery post-chemotherapy. Patients with missing/unknown race or ethnicity (*N* < 11), grade (*N* = 16), or tumor response (*N* = 181) were excluded. Patient characteristics for those excluded due to unknown tumor response are reported in Supplemental Table 1. This project was reviewed by our Institutional Review Board and determined not to be human subjects research because of the deidentified data and minimal risk to patients.

### Variables of interest

The outcome of interest was pathologic response to neoadjuvant therapy. We derived this outcome from NAACCR data elements 2875 (before 2018) and 3922 (2018 and after), which records the physician’s statement of response to neoadjuvant chemotherapy. Response categories recorded in the tumor registry data included complete response, partial response, response not otherwise specified (NOS; response noted but no mention if complete or partial), no response, and unknown or missing response. For some patients with unknown or missing response (*N* = 107), we were able to calculate response by comparing the post-therapy pathologic staging variables (ypT, ypN, ypM) to clinical TNM staging variables (AJCC 7th edition). For analysis, we dichotomized the outcome into pCR vs. non-pCR. Patients with partial response and no response were categorized as non-pCR. Patients with “response NOS” noted were conservatively classified as partial response and thus also included in the non-pCR group.

Cytotoxic chemotherapy regimen was the primary independent variable of interest; we compared the two-drug regimen of carboplatin plus taxane (CbT) to the four-drug regimen of doxorubicin plus cyclophosphamide and carboplatin plus taxane (AC-CbT). Receipt of pembrolizumab (yes or no) was also measured. Patient demographic variables and tumor characteristics gathered from the tumor table included age at diagnosis (< 40, 40–49, 50–59, and 60+), race (white, black, and other), ethnicity (Hispanic or not), stage (I–II or III), and grade (1–2 or 3). The most recent height and weight measurement prior to initiating chemotherapy was extracted from the EHR data to calculate body mass index (BMI). Comorbidity weights were calculated using the National Cancer Center (NCI) SAS Macro (2021 version) with breast-specific weights [27].

### Statistical analysis

After examining carboplatin uptake in the overall TNBC cohort, all subsequent analyses were restricted to those in the two treatment groups. Descriptive statistics were used to summarize baseline characteristics across these treatment groups. We used multivariable logistic regression with inverse probability weighting and robust standard errors to estimate the response to neoadjuvant therapy associated with treatment regimen while adjusting for demographic, tumor, and treatment-related and health status variables described above. Treatment probabilities were estimated using logistic regression, and separate models were estimated for patient subsamples diagnosed before or after August 2021 to account for dramatic changes in treatment selection patterns that followed the introduction of pembrolizumab. Indicator variables for GPC network site were also included to account for geographic variation in treatment patterns and patient characteristics across participating sites. To assess the robustness of our findings to outcome misclassification, we conducted two sensitivity analyses, one in which all “response NOS” were classified as pCR and another dropping patients with “response NOS” from the analytic sample.

## Results

**Fig. 1:**
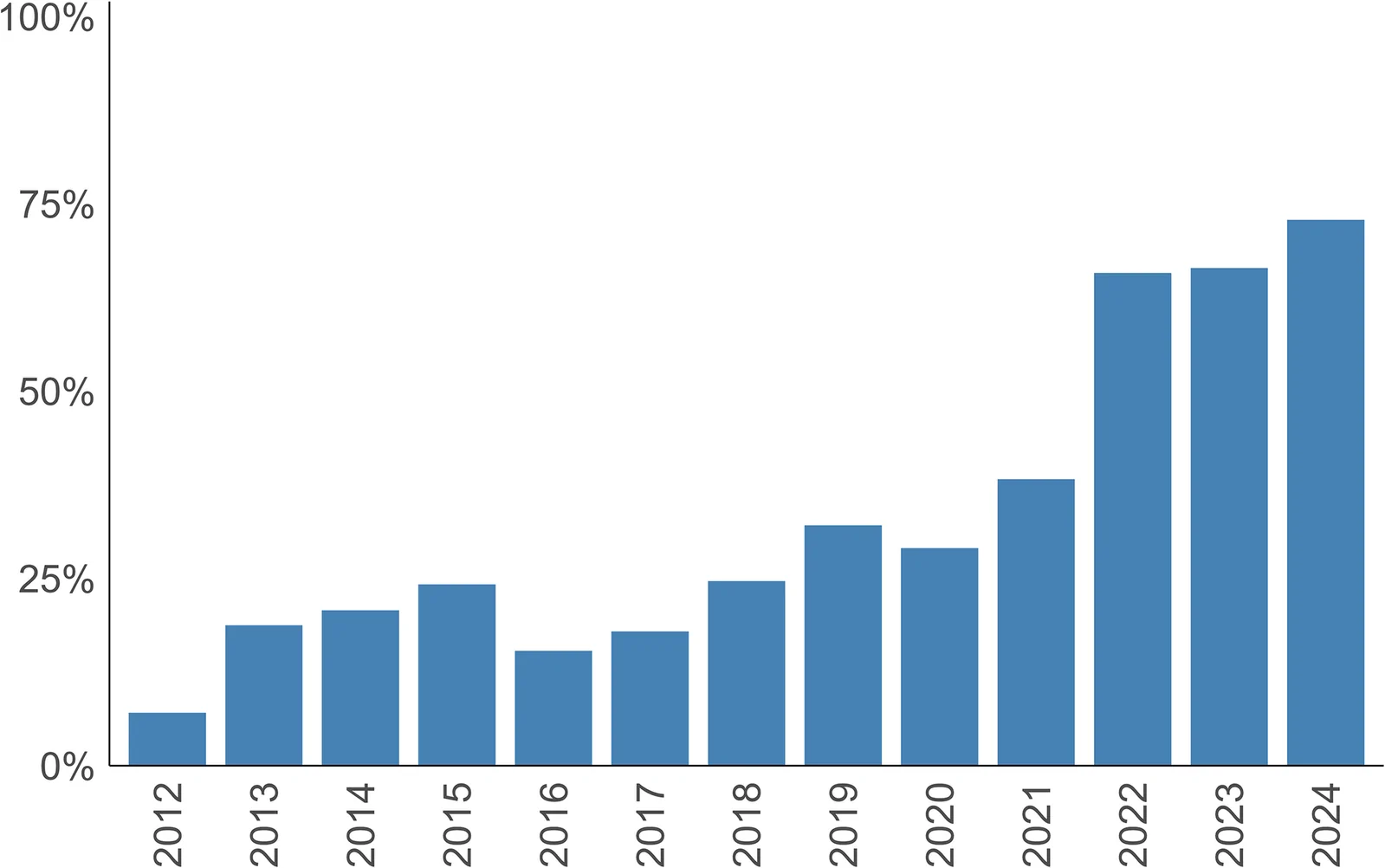
Percent of TNBC patients (*N* = 2,413) receiving carboplatin-containing regimen by diagnosis year

There were 2,413 early-stage TNBC patients in the data. The proportion of TNBC patients receiving any carboplatin-containing regimen increased dramatically over the study period, from 7.1% in 2012 to 72.8% in 2024 (Fig. 1).

### Demographic and clinical characteristics overall

**Fig. 2:**
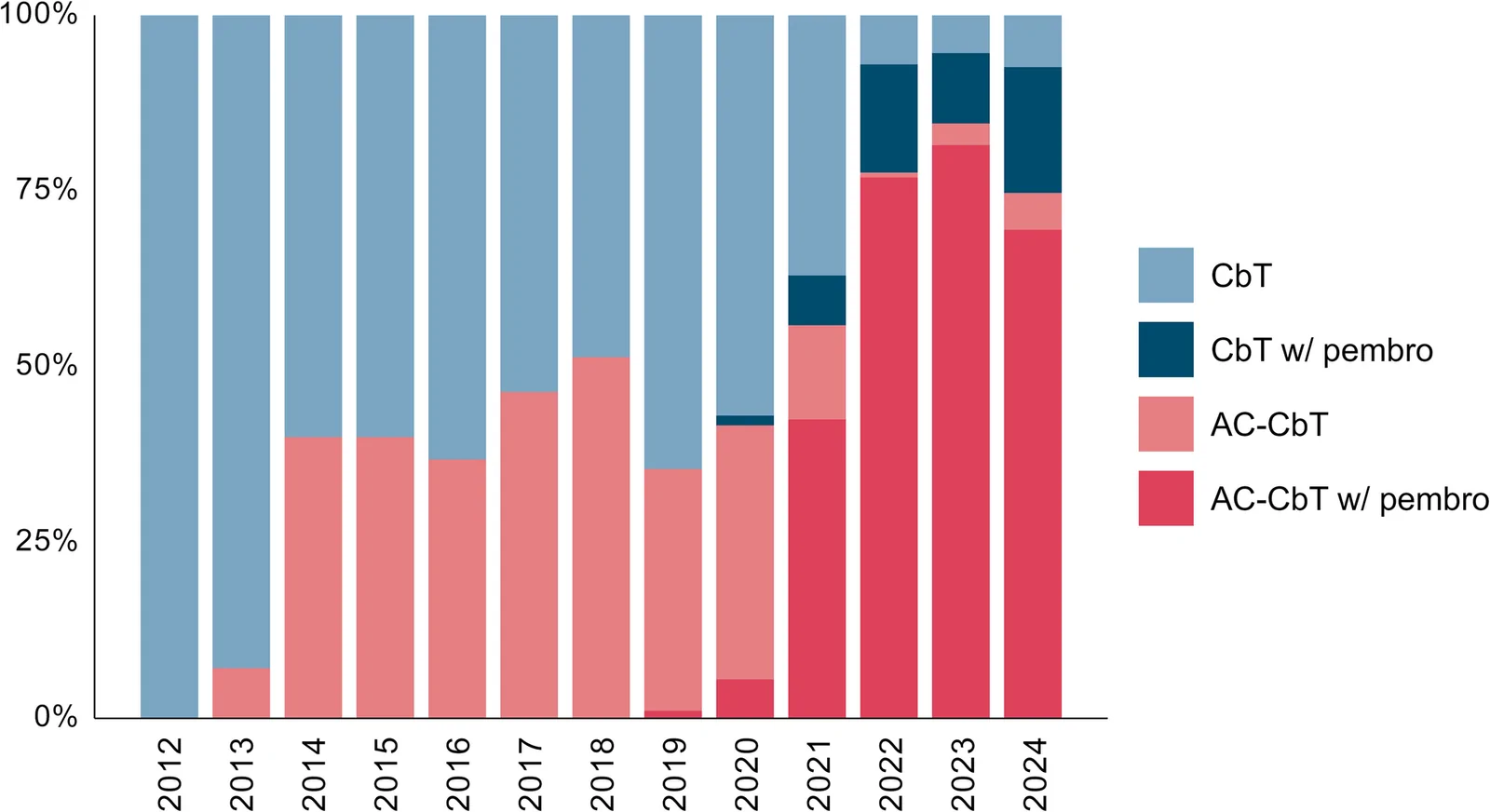
Proportion of TNBC patients receiving CbT and AC-CbT, with and without pembrolizumab, by diagnosis year (*N* = 878). Abbreviations. CbT = carboplatin + taxane. AC-CbT = carboplatin + taxane followed by anthracycline + cyclophosphamide. pembro = pembrolizumab

The final study sample included 878 TNBC patients, 362 (41.2%) receiving CbT and 516 (58.8%) receiving AC-CbT (Table 1). Almost half received pembrolizumab, with swift uptake starting in 2021 (Fig. 2). The mean (standard deviation [SD]) age at diagnosis was 51.2 (13.06) and 15.9% presented with stage I disease. High-grade tumors were common, with 87.5% classified as grade 3. Mean (SD) BMI was 30.19 (7.37), with 10.4% categorized as Class 3 Obesity. The majority of patients had no comorbidity at time of diagnosis (91.9%), and mean (SD) NCI comorbidity index was low, 0.05 (0.21) [28].

**Table 1:** Baseline characteristics for TNBC patients receiving CbT or AC-CbT (*N* = 878), stratified by cytotoxic treatment regimen*

	Full Sample	CbT	AC-CbT	*p*-value
Sample size	878	362	516	
Got pembrolizumab				< 0.001
No	474 (54.0%)	300 (82.9%)	174 (33.7%)	
Yes	404 (46.0%)	62 (17.1%)	342 (66.3%)	
Age at diagnosis				< 0.001
< 40	201 (22.9%)	61 (16.9%)	140 (27.1%)	
40–49	216 (24.6%)	74 (20.4%)	142 (27.5%)	
50–59	197 (22.4%)	81 (22.4%)	116 (22.5%)	
60+	264 (30.1%)	146 (40.3%)	118 (22.9%)	
Mean (SD) age at diagnosis	51.2 (13.1)	54.7 (13.4)	47.7 (12.3)	< 0.001
Race				0.700
White	679 (77.3%)	275 (76.0%)	404 (78.3%)	
Black	149 (17.0%)	65 (18.0%)	84 (16.3%)	
Other	50 (5.7%)	22 (6.1%)	28 (5.4%)	
Hispanic				0.006
No	832 (94.8%)	**	480 (93.0%)	
Yes	46 (5.2%)	< 11**	36 (7.0%)	
Stage				< 0.001
I	140 (15.9%)	81 (22.4%)	59 (11.4%)	
II	412 (46.9%)	186 (51.4%)	226 (43.8%)	
III	326 (37.1%)	95 (26.2%)	231 (44.8%)	
Grade				0.200
1, 2	110 (12.5%)	51 (14.1%)	59 (11.4%)	
3	768 (87.5%)	311 (85.9%)	457 (88.6%)	
BMI, categorical				0.100
Normal	240 (27.3%)	90 (24.9%)	150 (29.1%)	
Overweight	254 (28.9%)	100 (27.6%)	154 (29.8%)	
Class 1 Obesity	174 (19.8%)	86 (23.8%)	88 (17.1%)	
Class 2 Obesity	119 (13.6%)	45 (12.4%)	74 (14.3%)	
Class 3 Obesity	91 (10.4%)	41 (11.3%)	50 (9.7%)	
BMI, continuous				0.093
Min, Max	15.47, 57.96	15.97, 57.96	15.47, 56.91	
Mean (SD)	30.19 (7.37)	30.53 (7.18)	29.94 (7.50)	
NCI Comorbidity Index, categorical				0.091
No other comorbidity	807 (91.9%)	326 (90.1%)	481 (93.2%)	
Any comorbidity	71 (8.1%)	36 (9.9%)	35 (6.8%)	
NCI Comorbidity Index, continuous				0.087
Min, Max	0.00, 2.10	0.00, 2.10	0.00, 1.32	
Mean (SD)	0.05 (0.21)	0.07 (0.27)	0.04 (0.15)	

* Unless otherwise noted, values in parentheses reflect column percents

** In compliance with data use agreement, cell sizes less than 11 are suppressed

Abbreviations. CbT = carboplatin + taxane. AC-CbT = carboplatin + taxane followed by anthracycline + cyclophosphamide. SD = standard deviation. NCI = National Cancer Institute

### Demographic and clinical characteristics by treatment group

Compared to the CbT group, the AC-CbT group was younger on average, 47.7 (12.3) vs. 54.7 (13.4), and less likely to have any comorbidity (6.8% vs. 9.9%). They were also more likely to get pembrolizumab (66.3% vs. 17.1%), present with stage III disease (44.8% vs. 26.2%), and be normal weight (29.1% vs. 24.9%) – although there was little difference in mean BMI between the treatment groups. The distribution of race and grade were similar between the two treatment groups.

### Response to chemotherapy

Half of the sample achieved pCR (50.0%), 5.9% had no response to neoadjuvant chemotherapy, and 15.9% had response NOS (Table 2). pCR was observed in 47.2% and 51.9% of the CbT and AC-CbT groups, respectively. Receipt of pembrolizumab was associated with a higher rate of pCR (52.5% vs. 47.9%). The highest rate of pCR was observed in patients receiving AC-CbT with pembrolizumab (54.1%). Women diagnosed at age 40–49 years old (yo) were more likely to achieve pCR than any other age group (62.0% vs. 47.8%, 43.7%, and 46.6%).

**Table 2:** Response to neoadjuvant chemotherapy by treatment and baseline patient characteristic in TNBC patients receiving CbT or AC-CbT (*N* = 878)*

	Complete	Partial	Response NOS	No Response
Sample size	439 (50.0%)	247 (28.1%)	140 (15.9%)	52 (5.9%)
Cytotoxic regimen				
CbT	171 (47.2%) [39.0%]	100 (27.6%) [40.5%]	62 (17.1%) [44.3%]	29 (8.0%) [55.8%]
AC-CbT	268 (51.9%) [61.0%]	147 (28.5%) [59.5%]	78 (15.1%) [55.7%]	23 (4.5%) [44.2%]
Got pembrolizumab				
No	227 (47.9%) [51.7%]	139 (29.3%) [56.3%]	77 (16.2%) [55.0%]	31 (6.5%) [59.6%]
Yes	212 (52.5%) [48.3%]	108 (26.7%) [43.7%]	63 (15.6%) [45.0%]	21 (5.2%) [40.4%]
Age at diagnosis (yo)				
< 40	96 (47.8%) [21.9%]	65 (32.3%) [26.3%]	28 (13.9%) [20.0%]	**
40–49	134 (62.0%) [30.5%]	47 (21.8%) [19.0%]	26 (12.0%) [ ** ]	< 11**
50–59	86 (43.7%) [19.6%]	60 (30.5%) [24.3%]	39 (19.8%) [27.9%]	12 (6.1%) [23.1%]
60–65	123 (46.6%) [28.0%]	75 (28.4%) [30.4%]	47 (17.8%) [33.6%]	19 (7.2%) [36.5%]
Race				
White	343 (50.5%) [78.1%]	192 (28.3%) [77.7%]	109 (16.1%) [77.9%]	35 (5.2%) [67.3%]
Black	73 (49.0%) [16.6%]	41 (27.5%) [16.6%]	**	**
Other	23 (46.0%) [5.2%]	14 (28.0%) [5.7%]	< 11**	< 11**
Hispanic				
No	411 (49.4%) [93.6%]	237 (28.5%) [ ** ]	134 (16.1%) [ ** ]	50 (6.0%) [ ** ]
Yes	28 (60.9%) [6.4%]	< 11**	< 11**	< 11**
Stage				
I	85 (60.7%) [19.4%]	27 (19.3%) [10.9%]	22 (15.7%) [15.7%]	6 (4.3%) [11.5%]
II	214 (51.9%) [48.7%]	111 (26.9%) [44.9%]	59 (14.3%) [42.1%]	28 (6.8%) [53.8%]
III	140 (42.9%) [31.9%]	109 (33.4%) [44.1%]	59 (18.1%) [42.1%]	18 (5.5%) [34.6%]
Grade				
1, 2	41 (37.3%) [9.3%]	41 (37.3%) [16.6%]	17 (15.5%) [12.1%]	11 (10.0%) [21.2%]
3	398 (51.8%) [90.7%]	206 (26.8%) [83.4%]	123 (16.0%) [87.9%]	41 (5.3%) [78.8%]
BMI, categorical				
Normal	112 (46.7%) [25.5%]	70 (29.2%) [28.3%]	43 (17.9%) [30.7%]	15 (6.3%) [28.8%]
Overweight	134 (52.8%) [30.5%]	69 (27.2%) [27.9%]	38 (15.0%) [27.1%]	13 (5.1%) [25.0%]
Class 1 Obesity	94 (54.0%) [21.4%]	46 (26.4%) [18.6%]	24 (13.8%) [ ** ]	< 11**
Class 2 Obesity	63 (52.9%) [14.4%]	33 (27.7%) [13.4%]	16 (13.4%) [ ** ]	< 11**
Class 3 Obesity	36 (39.6%) [8.2%]	29 (31.9%) [11.7%]	19 (20.9%) [ ** ]	< 11**
BMI, continuous				
Min, Max	15.97, 54.41	15.47, 57.96	17.31, 53.64	18.34, 51.25
Mean (SD)	30.01 (6.93)	30.55 (7.79)	30.00 (7.77)	30.49 (7.96)
Median (IQR)	28.89 (24.93, 34.25)	29.21 (24.62, 35.01)	28.71 (24.34, 34.95)	29.28 (24.08, 35.09)
NCI Comorbidity Index, categorical				
No other comorbidity	36 (50.7%) [8.2%]	19 (26.8%) [7.7%]	13 (18.3%) [9.3%]	< 11**
Any comorbidity	403 (49.9%) [91.8]	228 (28.3%) [92.3%]	127 (15.7%) [90.7%]	**
NCI Comorbidity Index, continuous				
Min, Max	0.00, 1.32	0.00, 2.10	0.00, 2.01	0.00, 1.71
Mean (SD)	0.04 (0.15)	0.05 (0.20)	0.08 (0.30)	0.07 (0.29)

* Unless otherwise noted, values in parentheses reflect row percents and values in square brackets reflect column percents

** In compliance with data use agreement, cell sizes less than 11 are suppressed

Abbreviations. NOS = not otherwise specified. CbT = carboplatin + taxane. AC-CbT = carboplatin + taxane followed by anthracycline + cyclophosphamide. NCI = National Cancer Institute. SD = standard deviation. yo = years old

**Fig. 3:**
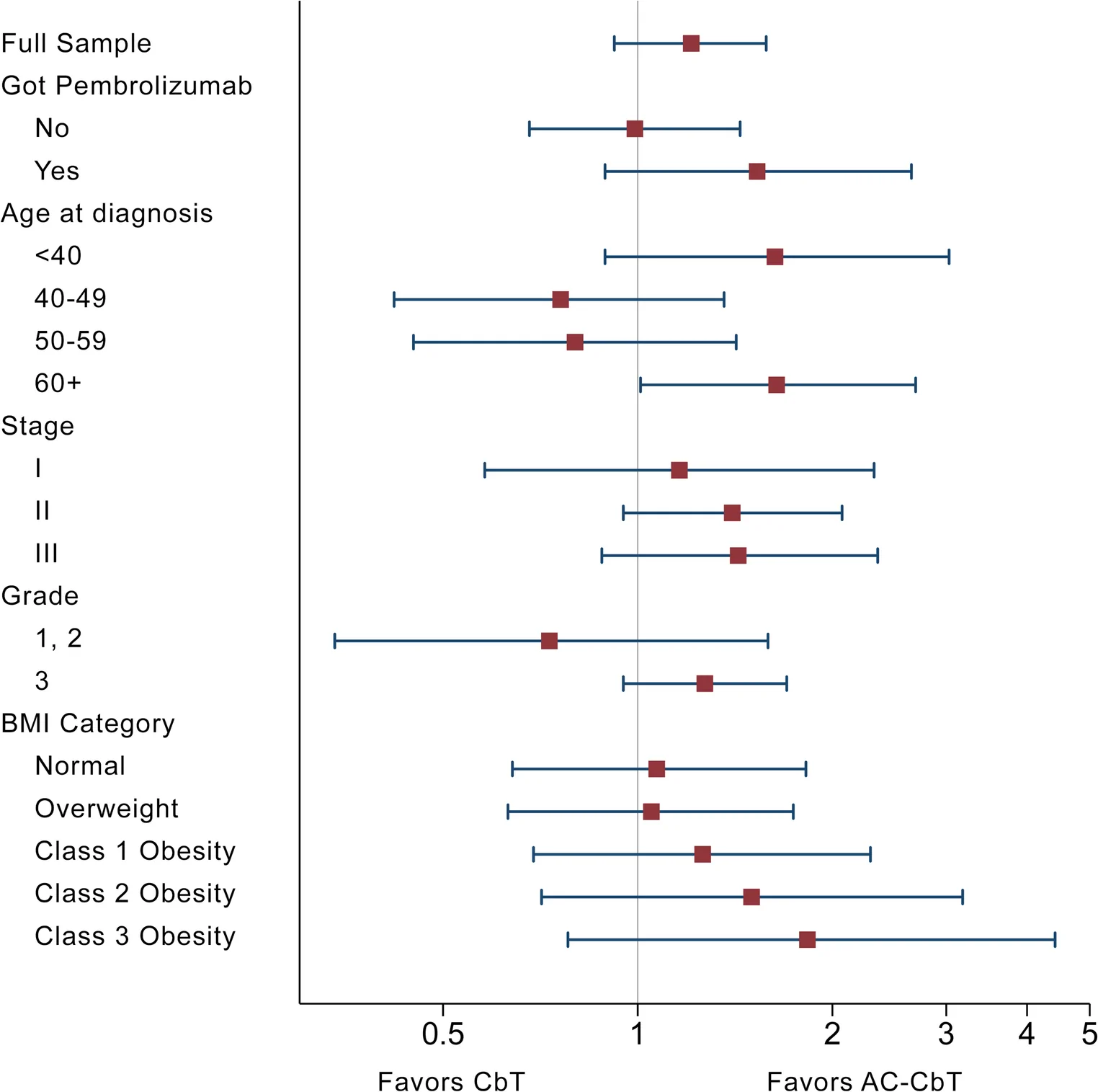
Odds of complete response following neoadjuvant chemotherapy in the AC-CbT vs. CbT group (*N* = 878), overall and stratified by patient clinical characteristics. Abbreviations. AC-CbT = carboplatin + taxane followed by anthracycline + cyclophosphamide. CbT = carboplatin + taxane

Overall, on univariate analysis, the AC-CbT group was more likely to achieve complete response than the CbT group, but this was not statistically different (odds ratio [OR] = 1.21, 95% confidence interval [CI] 0.92–1.58) on univariate analysis (Fig. 3), among patients who underwent surgery. In those who received pembrolizumab, the rate of pCR was higher in the AC-CbT group than the CbT group (54.1% vs. 43.5%), but not statistically different (OR = 1.53, 95%CI 0.89–2.65) among patients who underwent surgery. For patients 40–49 yo, the rate of pCR was lower in the AC-CbT group than the CbT group (59.9% vs. 66.2%) but not statistically different (OR = 0.76, 95%CI 0.42–1.36), among patients who underwent surgery.

Controlling for all other covariates, the odds of pCR did not differ between AC-CbT vs. CbT (adjusted OR [aOR] = 1.05, 95%CI 0.69–1.59) (Table 3), among patients who underwent surgery. Other factors were predictors of CR. Receipt of pembrolizumab was associated with pCR (aOR = 1.97, 95%CI 1.19–3.26), along with higher-grade disease (aOR = 1.84, 95%CI 1.07–3.16 for grade 3 vs. grades 1–2), among patients who underwent surgery. Post hoc analysis of an interaction term between receipt of AC-CbT and pembrolizumab was not statistically different (aOR = 1.09, 95%CI 0.46–2.62), among patients who underwent surgery. Stage was inversely related to CR, with lower odds of pCR in higher-stage disease. Patients diagnosed at age 40–49 yo were more likely to achieve pCR than the reference group, patients diagnosed 60+ (aOR = 2.09, 95%CI 1.34–3.27), among patients who underwent surgery. These findings were robust in sensitivity analyses. Categorizing patients with “response NOS” as pCR or dropping them from the analysis altogether resulted in similar estimates and the same conclusions (Supplemental Table 2). Pembrolizumab, higher grade, lower stage, and age 40–49 yo at diagnosis were all associated with higher odds of pCR, compared to their reference groups, and still no differences were observed between the AC-CbT vs. CbT groups (aOR = 0.94, 95%CI 0.61–1.45), among patients who underwent surgery.

**Table 3:** Inverse probability-weighted multivariable logistic regression model of factors associated with complete response in TNBC patients treated With CbT or AC-CbT (*N* = 878)*

	aOR	95%CI	*p*-value
Cytotoxic regimen			
CbT	(ref)		
AC-CbT	1.05	0.69–1.59	0.816
Got pembrolizumab			
No	(ref)		
Yes	1.97	1.19–3.26	0.008
Age at diagnosis (yo)			
< 40	0.99	0.62–1.56	0.956
40–49	2.09	1.34–3.27	0.001
50–59	1.07	0.66–1.73	0.781
60+	(ref)		
Race			
White	(ref)		
Black	1.09	0.70–1.69	0.706
Other	0.72	0.36–1.42	0.341
Hispanic			
No	(ref)		
Yes	2.28	1.15–4.53	0.019
Stage			
I	(ref)		
II	0.58	0.35–0.96	0.034
III	0.32	0.19–0.55	< 0.001
Grade			
1, 2	(ref)		
3	1.84	1.07–3.16	0.027
BMI	0.98	0.96–1.00	0.121
NCI Comorbidity Index	0.99	0.42–2.31	0.982

* Treatment site (*N* = 11) was also controlled for in all the above model but is omitted from the results for brevity

Abbreviations. aOR = adjusted odds ratio. 95%CI = 95% confidence interval. CbT = carboplatin + taxane. AC-CbT = carboplatin + taxane followed by anthracycline + cyclophosphamide. ref = reference group. NCI = National Cancer Institute. yo = years old

Post hoc descriptive analysis was conducted to further illuminate the finding of higher pCR rates in patients 40–49 yo at diagnosis. We found no evidence to indicate differences for this age group. There was an inverse relationship between use of AC-CbT and age at diagnosis, with utilization rates decreasing in each advancing age group (Supplemental Table 3). Similarly, the 40–49 yo cohort was more likely to receive pembrolizumab than the 50–59 yo cohort, but less likely to receive pembrolizumab than the < 40 yo cohort. No oddities were observed in the distribution of stage and grade across age groups. Receipt of pembrolizumab was stratified by cytotoxic chemotherapy regimen in addition to age at diagnosis (Supplemental Table 4, Supplemental Fig. 1), and we found no evidence that those 40–49 yo were more likely to receive pembrolizumab in either the Cb or AC-CbT group. No additional information was gained from post hoc descriptive analyses to explain the higher odds of pCR observed in the 40–49 yo group compared to the other age groups. Classification trees (single decision tree, conditional inference tree, random forest, XGBoost, and lightGBM) were also fitted, and none of the results from these non-parametric models suggested selection bias in the treatment choice (results not shown).

## Discussion

This is, to our knowledge, the largest observational study of a TNBC cohort spanning the immunotherapy era. We report a dramatic rise in carboplatin and pembrolizumab use during this period, reflecting the rapid changes in practice. Our study sample included 878 real-world patients diagnosed 2012–2024 with early-stage TNBC and treated with CbT or AC-CbT in 10 sites across the United States, among which 50.0% achieved pCR. As expected, pembrolizumab was associated with pCR. In contrast, we observed no improvement in pCR rates with the addition of anthracyclines to carboplatin-based regimens. This finding was robust across univariate and multivariate analyses.

This study adds important information regarding outcomes of the rapidly evolving TNBC treatment options. Management of early-stage TNBC shifted substantially in 2021 following publication of the KEYNOTE-522 trial, which established perioperative pembrolizumab combined with cytotoxic chemotherapy as the standard of care for stage II–III disease. However, the complex interaction between immunotherapy and cytotoxic chemotherapy is not fully understood and the assumption that benefit increases proportionately with number of agents may not apply with immunochemotherapy. In our study, the odds of pCR did not differ between the CbT and AC-CbT groups, controlling for pembrolizumab and other covariates. In the non-comparative, Phase II Neo-N trial, pCR rates of 67% and 71% were reported with carboplatin, paclitaxel, and nivolumab in a subset of biomarker-selected patients [29]; rates comparable to KEYNOTE-522 and free of anthracyclines. Although support for de-escalation of therapy for TNBC continues to be a topic of commentaries and editorials [30–32], it is still a challenge for oncologists treating this aggressive disease [33]. We eagerly await results from the ongoing SCARLET trial [34] evaluating whether anthracyclines can be safely omitted from the KEYNOTE-522 regimen while maintaining noninferior efficacy and reducing anthracycline-associated toxicities.

Although we report higher odds of pCR with pembrolizumab, the absolute rates of pCR in our study are lower than what is reported in KEYNOTE-522 (54.1% vs. 64.8% in the AC-CbT with pembrolizumab group) [17]. This may be partly due to differences between our cohort and those enrolled in trials (e.g., older, more frail, sicker, complete treatment less frequently) [35]. Additionally, 15.9% of our sample were noted as “response NOS” in the tumor registry data, which we conservatively categorized as non-pCR. The true rate is likely higher and may be closer to what was observed in the clinical trials [35].

Oncologists use the National Comprehensive Cancer Network (NCCN) Clinical Practice Guidelines in Oncology as evidence-based, consensus-driven tools to guide treatment decisions, with studies showing that as many as 95% of US oncologists rely on them in clinical practice [36]. AC-CbT without pembrolizumab and CbT with pembrolizumab have never been listed in guidelines, yet 27% of our sample received these regimens. In our study, patients at higher risk of adverse events and toxicity—proxied by older age and increased comorbidity—were more likely to forgo anthracyclines and receive CbT only. On the other hand, those with more aggressive disease—proxied by higher stage and grade—were more likely to receive all four cytotoxic drug classes, AC-CbT. This suggests that oncologists intentionally balance the risks and benefits of treatment in real-world practice, tailoring regimen selection to provide patient-centered care.

Our study shows that carboplatin has been adopted in stage I patients in real-world practice, despite their exclusion from KEYNOTE-522 and current NCCN guidelines, which list AC-T as the preferred regimen for stage I disease. In the larger sample of all TNBC patients diagnosed 2012–2024 with stage I disease, 19.4% received carboplatin as part of their chemotherapy regimen, with utilization increasing dramatically over time: 3.4% in 2012 to 12.1% in 2018 and 41.7% in 2024. Odds of pCR were similar between CbT vs. AC-CbT in our stage I patients who underwent surgery (OR = 1.16, 95%CI 0.58–2.32), bringing into question the possibility of substituting carboplatin/taxane combinations for sequential anthracycline/taxane regimens. There is precedence for this strategy in the adjuvant setting; the adjuvant PATTERN trial, where 74% of enrolled patients were node-negative, reported no difference in 5-year OS with carboplatin plus paclitaxel vs. an anthracycline- and taxane-based regimen [37].

Unexpectedly, we found patients 40–49 yo at diagnosis were more likely to have pCR. This difference was robust in univariate and multivariate and sensitivity analyses that controlled for other covariates. Post hoc analyses did not indicate major issues in selection bias or confounding associated with this age group. It is not clear from our data why this group did better than women diagnosed at other ages. Recent Phase III trials report an age effect, although the results are contradictory, and the racial composition of the enrolled population differs from our sample. In the Chinese RJBC 1501 trial of 786 women with TNBC, patients age ≤ 50 had worse disease-free survival than women 50+ (p-value = 0.001 for interaction), and no benefit with the addition of carboplatin [38]. In contrast, an Indian trial enrolling 720 women with TNBC found patients age ≤ 50 had better event-free survival than women 50+ (p-value = 0.01 for interaction), and a survival benefit with the addition of carboplatin [39]. Additional research is needed to clarify the effect and interaction of age on outcomes.

There are limitations to this study. First, reliance on EHR‑derived data introduces the possibility of incomplete or inconsistently captured clinical information, especially with multi-site data. Second, the cohort reflects limited racial and ethnic diversity, which may restrict the generalizability of findings to broader populations. Third, although this represents the largest real‑world analysis of its kind, the study spans a limited number of years in the immunotherapy era. Fourth, its observational design may be influenced by unmeasured confounding. Fifth, manual data abstraction on the part of the hospital tumor registries carries a risk of abstractor error, particularly in the classification of subtype and treatment response, and the presence of a non‑trivial number of “response not otherwise specified (NOS)” cases further limits precision. However, each of the sites within the GPC network are American College of Surgeons Commission on Cancer (CoC)-accredited and are required to submit high-quality tumor registry data. Sixth, key clinical variables such as performance status and BRCA status were not available, reducing our ability to adjust for important prognostic factors. Finally, we lacked information on toxicity, adherence, and relative dose intensity (RDI). The lack of benefit observed in this study with anthracyclines could be explained by lower RDI in the AC-CbT group. Even so, the purpose of this retrospective observational study was to assess real-world utilization and effectiveness between the CbT and AC-CbT regimens. Inability to tolerate the full guideline-recommended dose should be a factor in clinical decision-making.

## Conclusion

In this large, multi-site, real-world cohort of women with early-stage TNBC treated across the immunotherapy era, the addition of anthracyclines to a carboplatin-based neoadjuvant regimen was not associated with odds of pathologic CR. This finding was robust across sensitivity analyses and was consistent regardless of pembrolizumab use or disease stage. Our data also demonstrate the rapid and broad adoption of carboplatin-containing regimens in real-world practice, including off-guideline use in stage I disease. Together, these findings support clinical equipoise regarding anthracycline omission in early-stage TNBC and suggest that carboplatin-based, anthracycline-free regimens may offer comparable oncologic efficacy with a more favorable toxicity profile for many patients. Future observational research should evaluate the relative dose intensity of chemotherapy received, as well as rates of toxicity and long-term survival outcomes. Finally, data from the ongoing trials, such as the SCARLET trial [34], will be essential to definitively characterize the benefit of anthracycline omission in the contemporary immunotherapy era and inform future development of treatment guidelines.

## Supplementary Information

Below is the link to the electronic supplementary material.

Supplementary Material 1

## Data Availability

Data utilized in this study was accessed through the Greater Plains Collaborative (GPC) Reusable Observable Unified Study Environment (GROUSE). While data will not be available on public data repositories, investigators interested using the data can inquire through the GPC Front Porch (https://gpcnetwork.org/collaboration/).
