# Supplementary material for: Anthracycline-containing versus anthracycline-free carboplatin-based regimens in early-stage triple-negative breast cancer: real-world outcomes
Source: Breast Cancer Res Treat. 2026 Sep 9;219(2):7. doi: 10.1007/s10549-026-08071-8 (PMC13558311; doi:10.1007/s10549-026-08071-8)
Supplement: Supplementary file 1 [file 10549_2026_8071_MOESM1_ESM.docx]

**Supplemental Table 1.** Baseline Characteristics for TNBC Patients Excluded from Final Study Cohort Due to Missing Tumor Response Information

|  |  | **Sample (N=181)** |
| --- | --- | --- |
| Cytotoxic Regimen | |  |
|  | CbT | 47 (26.0%) |
|  | AC-CbT | 134 (74.0%) |
| Got pembrolizumab | |  |
|  | No | 118 (65.2%) |
|  | Yes | 63 (34.8%) |
| Age at diagnosis | |  |
|  | <40 | 38 (21.0%) |
|  | 40-49 | 46 (25.4%) |
|  | 50-59 | 43 (23.8%) |
|  | 60+ | 54 (19.8%) |
| Mean (SD) age at diagnosis | | 51.2 (13.1) |
| Race | |  |
|  | White | 149 (82.3%) |
|  | Black | 25 (13.8%) |
|  | Other | 7 (3.9%) |
| Hispanic | |  |
|  | No | 172 (95.0%) |
|  | Yes | 9 (5.0%) |
| Stage | |  |
|  | I | 31 (17.1%) |
|  | II | 90 (49.7%) |
|  | III | 60 (33.1%) |
| Grade | |  |
|  | 1, 2 | 25 (13.8%) |
|  | 3 | 156 (86.2%) |
| BMI, categorical | |  |
|  | Normal | 54 (29.8%) |
|  | Overweight | 52 (28.7%) |
|  | Class 1 Obesity | 37 (20.4%) |
|  | Class 2 Obesity | 19 (10.5%) |
|  | Class 3 Obesity | 19 (10.5%) |
| BMI, continuous | |  |
|  | Min, Max | 17.36, 54.20 |
|  | Mean (SD) | 29.85 |
| NCI Comorbidity Index, categorical | |  |
|  | No other comorbidity | 23 (12.7%) |
|  | Any comorbidity | 158 (87.3%) |
| NCI Comorbidity Index, continuous | |  |
|  | Min, Max | 0.00, 0.89 |
|  | Mean (SD) | 0.03 |

**Supplemental Table 2.** Sensitivity Analyses for Inverse Probability-Weighted Multivariable Logistic Regression Model, Categorizing Response NOS as Complete Response or Dropped From the Analysis, in TNBC Patients Receiving CbT or AC-CbT*

|  |  | **NOS categorized as “Complete Response” (N=878)** | | **NOS dropped from analysis (N=738)** | |
| --- | --- | --- | --- | --- | --- |
|  |  | **aOR** | **95%CI** | **aOR** | **95%CI** |
| Cytotoxic regimen | |  |  |  |  |
|  | CbT | (ref) |  | (ref) |  |
|  | AC-CbT | 0.94 | 0.61–1.45 | 0.97 | 0.62–1.51 |
| Got pembrolizumab | |  |  |  |  |
|  | No | (ref) |  | (ref) |  |
|  | Yes | 1.73 | 1.07–2.79 | 2.52 | 1.45–4.37 |
| Age at diagnosis (yo) | |  |  |  |  |
|  | <40 | 0.85 | 0.52–1.37 | 0.87 | 0.52–1.43 |
|  | 40-49 | 1.91 | 1.19–3.08 | 2.14 | 1.31–3.48 |
|  | 50-59 | 1.24 | 0.77–1.99 | 1.19 | 0.73–1.95 |
|  | 60+ | (ref) |  | (ref) |  |
| Race | |  |  |  |  |
|  | White | (ref) |  | (ref) |  |
|  | Black | 1.02 | 0.65–1.59 | 1.04 | 0.65–1.67 |
|  | Other | 0.78 | 0.39–1.56 | 0.68 | 0.31–1.45 |
| Hispanic | |  |  |  |  |
|  | No | (ref) |  | (ref) |  |
|  | Yes | 1.67 | 0.78–3.56 | 2.31 | 1.04–5.13 |
| Stage | |  |  |  |  |
|  | I | (ref) |  | (ref) |  |
|  | II | 0.64 | 0.36–1.13 | 0.52 | 0.28–0.93 |
|  | III | 0.43 | 0.24–0.77 | 0.31 | 0.16–0.57 |
| Grade | |  |  |  |  |
|  | 1, 2 | (ref) |  | (ref) |  |
|  | 3 | 1.78 | 1.05–3.02 | 2.31 | 1.32–4.03 |
| BMI | | 0.97 | 0.95–1.00 | 0.97 | 0.95–1.00 |
| NCI Comorbidity Index | | 1.22 | 0.50–3.02 | 1.40 | 0.49–4.02 |

* Treatment site (N=11) was also controlled for in all the above model but is omitted from the results for brevity.

Abbreviations. NOS = not otherwise specified. aOR = adjusted odds ratio. 95%CI = 95% confidence interval. CbT = carboplatin + taxane. AC-CbT = carboplatin + taxane followed by anthracycline + cyclophosphamide. ref = reference group. NCI = National Cancer Institute. yo = years old.

**Supplemental Table 3.** Select Baseline Characteristics, Stratified by Age at Diagnosis, in TNBC Patients Receiving CbT or AC-CbT (N=878)*

|  |  | **<40 (N=201)** | **40-49 (N=216)** | **50-59 (N=197)** | **60+ (N=264)** |
| --- | --- | --- | --- | --- | --- |
| Cytotoxic regimen | |  |  |  |  |
|  | CbT | 61 [30.3%] | 74 [34.3%] | 81 [41.1%] | 146 [55.3%] |
|  | AC-CbT | 140 [69.7%] | 142 [65.7%] | 116 [58.9%] | 118 [44.7%] |
| Got pembrolizumab | |  |  |  |  |
|  | No | 95 [47.3%] | 122 [56.5%] | 117 [59.4%] | 140 [53.0%] |
|  | Yes | 106 [52.7%] | 94 [43.5%] | 80 [40.6%] | 124 [47.0%] |
| Stage | |  |  |  |  |
|  | I | 26 [12.9%] | 31 [14.4%] | 35 [17.8%] | 48 [18.2%] |
|  | II | 83 [41.3%] | 99 [45.8%] | 89 [45.2%] | 141 [53.4%] |
|  | III | 92 [45.8%] | 86 [39.8%] | 73 [37.1%] | 75 [28.4%] |
| Grade | |  |  |  |  |
|  | 1, 2 | 20 [10.0%] | 24 [11.1%] | 20 [10.2%] | 46 [17.4%] |
|  | 3 | 181 [90.0%] | 192 [88.9%] | 177 [89.8%] | 218 [82.6%] |

* Values in square brackets reflect column percents.

Abbreviations. CbT = carboplatin + taxane. AC-CbT = carboplatin + taxane followed by anthracycline + cyclophosphamide.

**Supplemental Table 4.** Receipt of Pembrolizumab and Tumor Response, Stratified by Age at Diagnosis and Cytotoxic Chemotherapy Regimen, in TNBC Patients Receiving CbT or AC-CbT (N=878)*

|  |  | **<40** | | **40-49** | | **50-59** | | **60+** | |
| --- | --- | --- | --- | --- | --- | --- | --- | --- | --- |
|  |  | **CbT N=61** | **AC-CbT N=140** | **CbT N=74** | **AC-CbT N=142** | **CbT N=81** | **AC-CbT N=116** | **CbT N=146** | **AC-CbT N=118** |
| Got pembrolizumab | |  |  |  |  |  |  |  |  |
|  | No | 50 [82.0%] | 45 [32.1%] | ** | 57 [40.1%] | ** | 43 [37.1%] | 111 [76.0%] | 29 [24.6%] |
|  | Yes | 11 [18.0%] | 95 [67.9%] | <11** | 85 [59.9%] | <11** | 73 [62.9%] | 35 [24.0%] | 89 [75.4%] |
| Tumor response | |  |  |  |  |  |  |  |  |
|  | pCR | 24 [39.3%] | 72 [51.4%] | 49 [66.2%] | 85 [59.9%] | 38 [46.9%] | 48 [41.4%] | 60 [41.1%] | 63 [53.4%] |
|  | non-pCR | 37 [60.7%] | 68 [48.6%] | 25 [33.8%] | 57 [40.1%] | 43 [53.1%] | 68 [58.6%] | 86 [58.9%] | 55 [46.6%] |

* Values in square brackets reflect column percents.

** In compliance with data use agreement, cell sizes less than 11 are suppressed.

Abbreviations. CbT = carboplatin + taxane. AC-CbT = carboplatin + taxane followed by anthracycline + cyclophosphamide. pCR = pathologic complete response.

**Supplemental Fig 1.** Proportion of patients receiving CbT and AC-CbT, with and without pembrolizumab, across years and stratified by age at diagnosis: <40 (A), 40–49 (B), 50–59 (C), 60+ (D). Abbreviations. CbT = carboplatin + taxane. AC-CbT = carboplatin + taxane followed by anthracycline + cyclophosphamide. pembro = pembrolizumab.
